# Fetal malformation in maternal toxoplasma and rubella co-infection in Cameroon: a case report

**DOI:** 10.1186/s13256-016-1133-y

**Published:** 2016-12-03

**Authors:** Andreas Ateke Njoh, Sarah Namondo Njoh, Messang Blandine Abizou

**Affiliations:** 1 Tombel District Hospital, Ministry of Public Health, Tombel, Republic of Cameroon; 2 Educational Psychology/Special Education, Faculty of Education, University of Buea, Buea, Republic of Cameroon; 3 General Medicine, Faculty of Medicine and Biomedical Sciences, University of Yaounde 1, Yaounde, Republic of Cameroon

**Keywords:** Toxoplasma and rubella co-infection, Anencephaly, Resource-limited settings, Case report

## Abstract

**Background:**

There has been a recent increase in the number of newborns with brain malformations due to congenital infections, but the impact of these diseases remains largely under ascertained in middle-income and low-income countries. This case report presents a fetal anencephaly following maternal toxoplasma and rubella co-infection in a resource-limited setting and the challenges faced by the patient and the health care provider in the management of the condition.

**Case presentation:**

A 25-year-old black Cameroonian woman of Bakossi origin, gravida_3_ para_1_010, presented with a positive rubella and toxoplasma immunoglobulin G serologic test at 21 weeks of pregnancy; she could not benefit from a fetal morphologic ultrasound partly because there was none at the site of her antenatal clinic and because there were accessibility constraints getting to the nearest referral hospital approximately 100 km away. She returned to the hospital in labor pains 14 weeks later and, upon examination, she was observed to be at almost full cervical dilatation and had a stillbirth a few minutes later; a baby boy weighing 1600 g with anencephaly.

The devastated parents of the baby were counseled and given psychological support. She was discharged from hospital 3 days later and now benefits from continual follow up as out-patient. She was advised to consult a gynecologist-obstetrician before her next pregnancy.

**Conclusion:**

Much attention still has to be paid to ameliorate the health care in resource-limited settings where pregnant women generally obtain less than adequate care.

**Electronic supplementary material:**

The online version of this article (doi:10.1186/s13256-016-1133-y) contains supplementary material, which is available to authorized users.

## Background

Birth defects are an urgent global health priority affecting millions of births worldwide, but the impact remains largely under ascertained in middle-income and low-income settings [1]. Neural tube defects such as anencephaly have been shown to have a multifactorial etiology, such as folic acid deficiency, genetic disorders, socioeconomic status, educational status, and exposure to a variety of environmental toxins [2, 3]. Congenital infections such as toxoplasmosis and rubella are known to play a non-negligible role in the development of brain malformations [1, 4].

To the best of our knowledge, most fetal abnormalities may go unreported in rural communities, like in the area of our report, where antenatal follow up routinely occurs away from health facilities with robust diagnostic capabilities. Also, in this setting, some cases may go unreported even in accredited health institutions.

This case report illustrates some of the challenges faced in delivering adequate health care to a pregnant woman with specialized needs in a resource-limited setting.

## Case presentation

We describe the case of a 25-year-old black Cameroonian woman of Bakossi origin with basic primary education, gravida 3 para 1 (G_3_P_1_010), who lost a child in 2012 following complications of neonatal infection and later had an abortion in early 2015. She presented to a district hospital in the South-West Region of Cameroon for her first antenatal visit with a 21-week pregnancy. Her blood pressure was 107/66 mmHg and she had a uterine fundal height of 26 cm.

She was requested to do some paraclinical examinations including blood group, hemoglobin level, glycemia, human immunodeficiency virus (HIV), syphilis, toxoplasma, rubella serology, stool analysis, urine analysis, and a fetal ultrasound. Most of these tests were done and were found to be normal. However, toxoplasma and rubella immunoglobulin G (IgG) serologic tests were both reactive; analysis was done with the aid of ImmunoComb® IgG and ImmunoComb® II IgG serologic tests, respectively. She also had a proteinuria of 100 mg/dl; her blood group is AB rhesus positive. She did not benefit from a morphologic fetal ultrasound partly because there was none in the hospital and because of the financial constraints she presented, which limited her movement to the nearest regional referral hospital located approximately 100 km from the site of her antenatal clinic via a poorly accessible road. She was, however, put on daily 65 mg of elemental iron and 5 mg of folic acid supplement, and she received anti-tetanus vaccine, intermittent preventive treatment against malaria, and a long-acting insecticide-treated bed net. She was encouraged to consult a gynecologist-obstetrician at the nearest referral hospital.

By her next antenatal visit 4 weeks later, she had not consulted the specialist physician and was still unable to attend the paraclinical examination requested earlier. Emphasis was placed on the risk of her baby sustaining life-threatening malformations and she was advised to continue with the supplements and follow-up visits. She was again encouraged to undergo a fetal ultrasound and to consult a gynecologist-obstetrician. Adding to the challenges faced by this expectant mother, the district hospital did not have an ambulance that could have helped the health care provider to overcome the road accessibility and financial challenges she faced.

During her 34th week of pregnancy she returned to the hospital in labor pains with a blood pressure of 110/68 mmHg, uterine fundal height of 40 cm, and was at 8 cm cervical dilation with bulging membranes. After placing her on a 5% glucose infusion, the membranes were ruptured, and a turbid amniotic fluid of approximately 2000 ml oozed out. This was followed by the delivery of an anencephalic recently dead baby boy weighing 1600 g. Active management of third stage of labor was done (Additional file 1).

The devastated mother and her partner received psychosocial care for 3 days; she was discharged from hospital and scheduled for routine psychosocial follow-up. She was further counseled on the need to consult a gynecologist-obstetrician before her next pregnancy.

## Discussion

Congenital infection caused by *Toxoplasma gondii* and rubella can cause serious damage to the newborn [1, 4] that can be diagnosed *in utero* or at birth [5–7]. Although these infections are rare in most developed countries, they still represent a major risk for pregnant women in developing regions [8]. Therefore, early diagnosis and treatment are vital to ensure proper management of infected persons.

It could be thought that this expectant mother was forced to carry a pregnancy with a fetus that was not viable to term partly because of her low educational status or limited resources. Worse, the district hospital, which is the main referral health institution in the sub-division where this client was followed up, lacks a functional ultrasound, which could have enabled early diagnosis of anencephaly *in utero* onsite to facilitate early management (an abortion) [9]. Furthermore, this district hospital did not have an ambulance that could have been used by the hospital administration to transport this patient to the nearest referral hospital located several kilometers away where she could have received more specialized care. This devastated mother, who has not been able to keep any of her children, will also need follow up from a genetic health care practitioner for her future pregnancies but, to the best of our knowledge, there are none in the South-West Region where this case is reported.

Unfortunately, due to the very limited resources in the setting of our report, it was not possible to ensure an early and proper management of the health problem of this patient. This challenge placed both the patient and the health care provider in great difficulty.

## Conclusions

Improving the health facility by providing the hospital with a functional ultrasound unit with proper staff training and an ambulance would go a long way in limiting similar challenges. This could improve the health management of the community.

## Additional file

Additional file 1: Photo of the stillborn baby with anencephaly at birth. (DOCX 502 kb)

## Abbreviations

GP: Gravidity parity
HIV: Human immunodeficiency virus
IgG: Immunoglobulin G
