# Supplementary figures and images for: Fetal malformation in maternal toxoplasma and rubella co-infection in Cameroon: a case report
Source: J Med Case Rep. 2016 Dec 3;10:345. doi: 10.1186/s13256-016-1133-y (PMC5135823; doi:10.1186/s13256-016-1133-y)

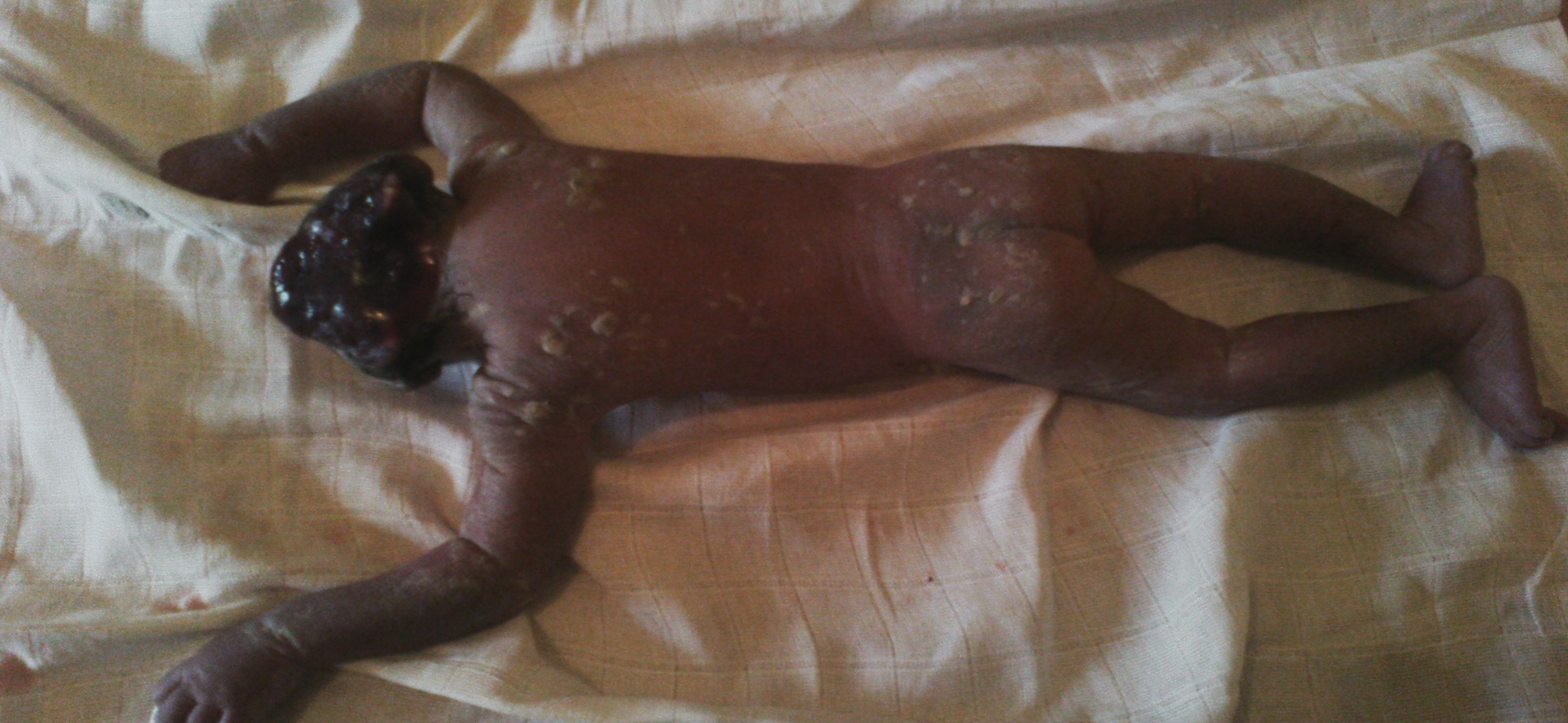


Photo of the Dead Anencephaly Baby at Birth

Supplement: Additional file 1: — Photo of the stillborn baby with anencephaly at birth. (DOCX 502 kb) [file 13256_2016_1133_MOESM1_ESM.docx]
